# Supplementary material for: Heritable functional architecture in human visual cortex
Source: Neuroimage. 2021 Oct 1;239:118286. doi: 10.1016/j.neuroimage.2021.118286 (PMC7611349; doi:10.1016/j.neuroimage.2021.118286)
Supplement: Supplementary file 1 [file mmc1.docx]

**Appendix: Supplementary Information**


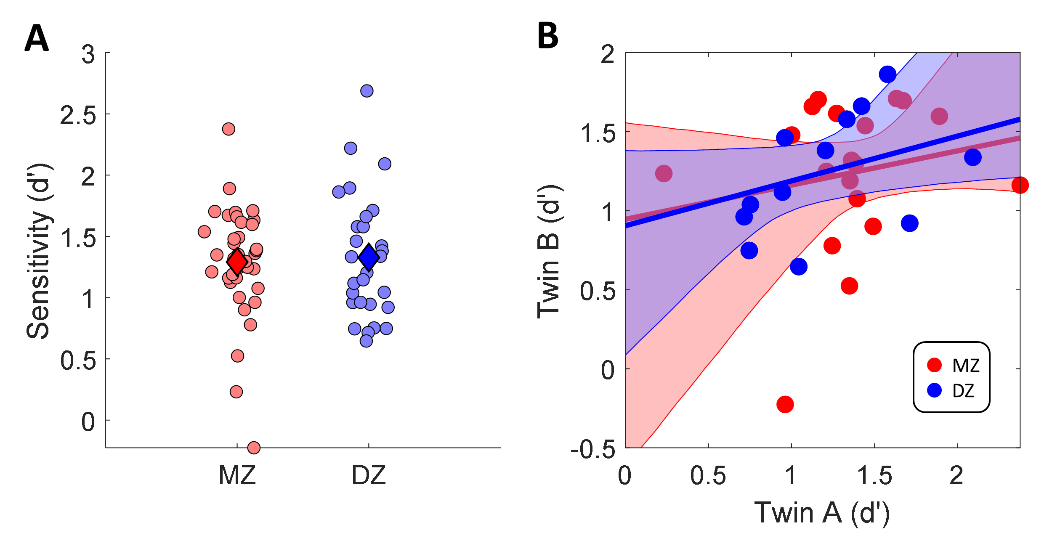


**Figure S1.** Behavioral results. A. Sensitivity (d’) on the behavioral task in the scanner for MZ and DZ twin groups. Each dot denotes the results for one participant in each group. Diamonds indicate the group means. B. Comparing behavioral sensitivity (d’) between twins in each pair. Each dot denotes a given twin pair. The solid lines denote the best fitting linear regression and the shaded regions shows the 95% confidence interval. Red: MZ twins. Blue: DZ twins.


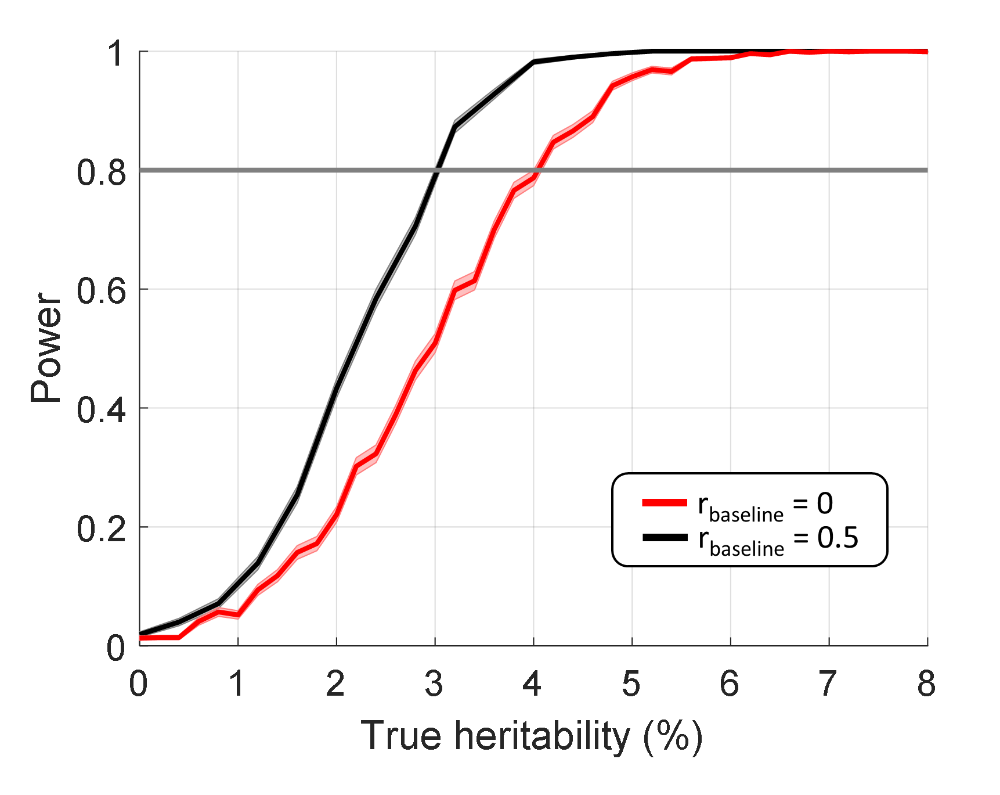


**Figure S2.** Power analysis for pRF parameter correlations. We simulated data with the same number of spatial points as the smallest visual region we report, area V3 (3409 vertices), and the same sample sizes for the MZ (n=19) and DZ (n=14) groups as in our study. We modulated the difference in correlation between the MZ and DZ groups to simulate a range of possible heritability values. We repeated this simulation 1,000 times. For each simulated heritability value, we then quantified the number of times that our bootstrapped heritability analysis (see Materials and Methods) detected a significant effect (p<0.05, Bonferroni corrected for 9 comparisons). The plot shows the detection rate (power) against true heritability. This suggests our analysis achieved 80% power to detect a true heritability of approximately 3-4% , depending on whether we assume a mid-range baseline intra-class correlation of r=0.5 or the minimal DZ correlation of r=0.


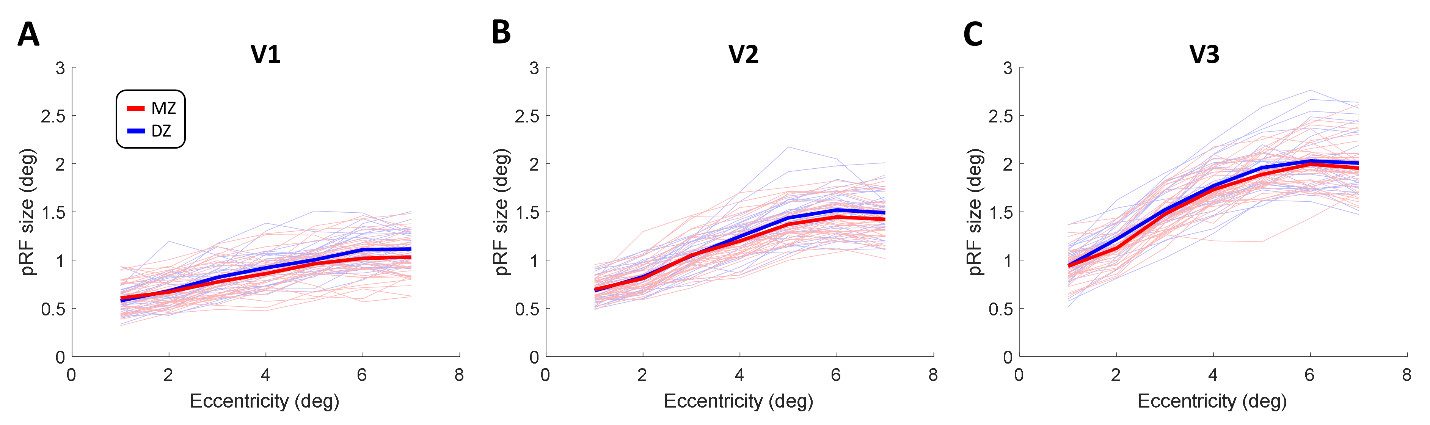


**Figure S3.** pRF size relative to eccentricity. pRF size data for each participant from V1 (A), V2 (B), or V3 (C) were binned into 1 degree wide eccentricity bins and averaged. Bin averages are plotted against eccentricity. The faint curves show individual participants. The thicker lines denote the mean of each twin group. Red: MZ, Blue: DZ.


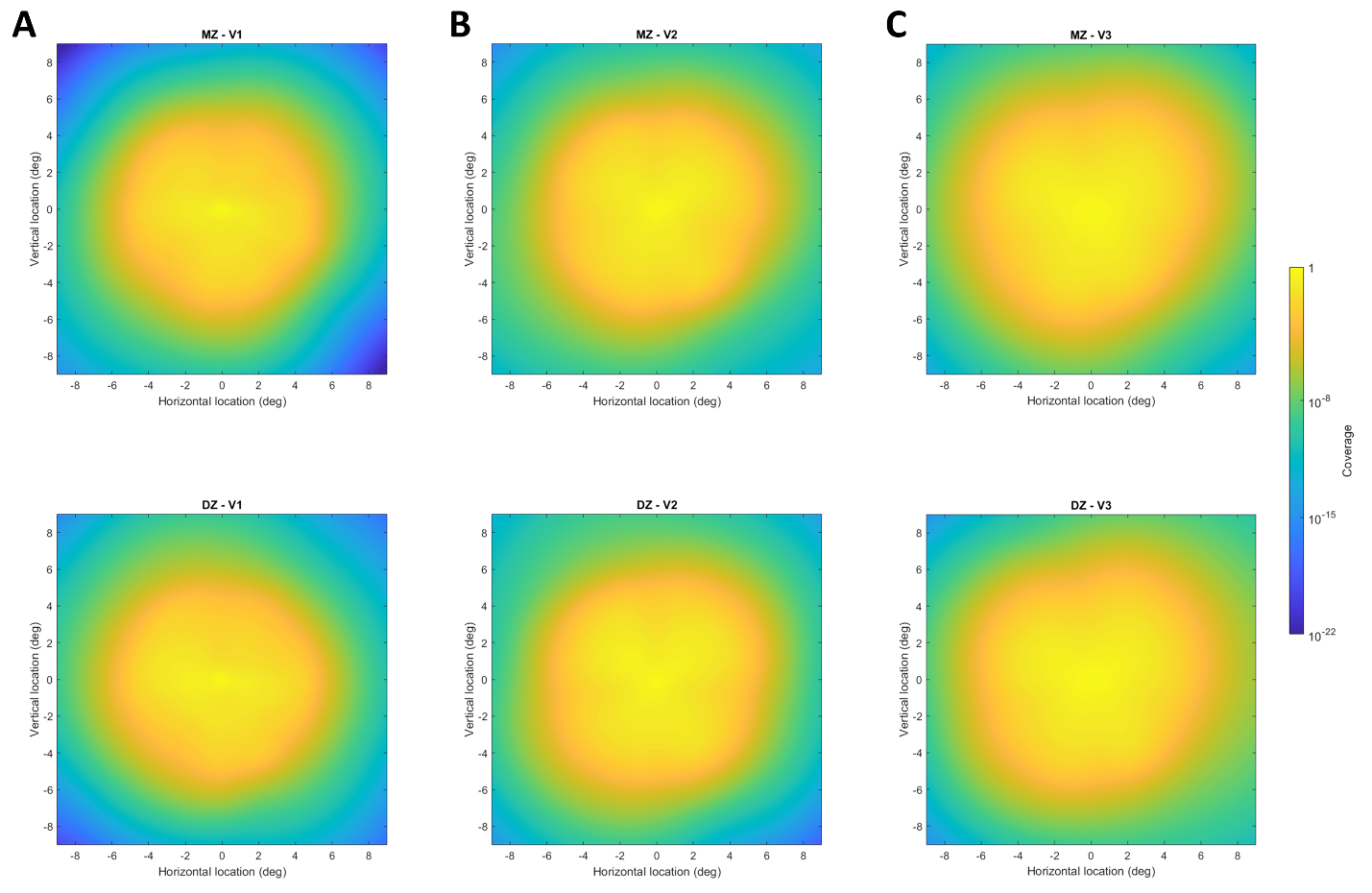


**Figure S4.**  Visual field coverage plots for V1 (A), V2 (B), and V3 (C). Visual field coverage reflect the average of all pRF profiles within a region, thresholded with R^2^>0.1 and only including pRFs whose centers fell within 9 degrees eccentricity. Plots were further averaged across all participants in each group. The color of each pixel denotes how densely each visual field location was sampled by the pRFs in the region, ranging from blue (low coverage) over green to yellow (high coverage). Top row: MZ twins. Bottom row: DZ twins.


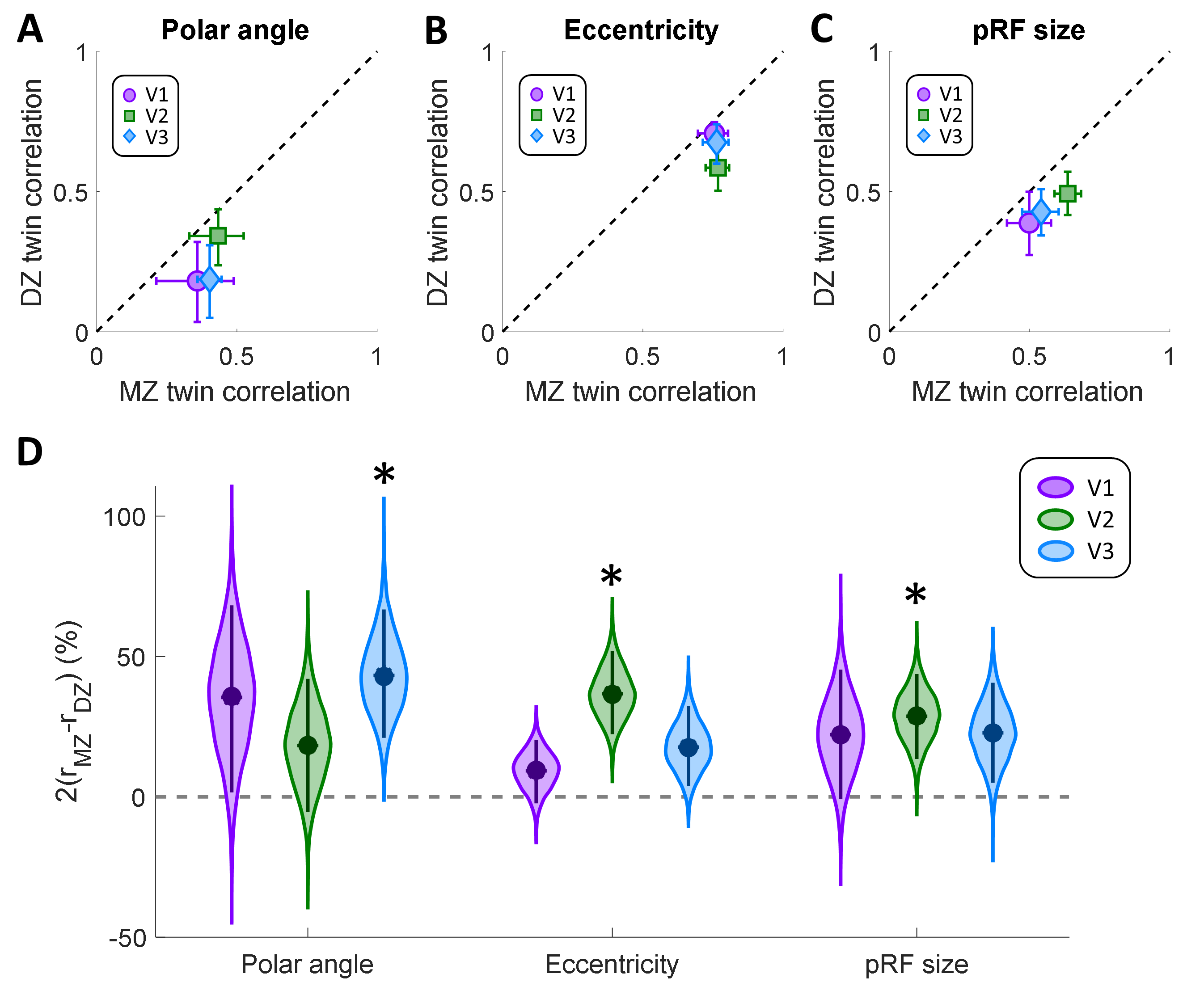


**Figure S5.** Control analysis of intra-class circular correlations for polar angle (A), and Spearman correlations for eccentricity (B), and pRF size (C). In order to provide an estimate that is less biased by the spatial dependence between neighboring vertices, we selected vertices with a minimal spatial separation of 8 mm between spatial sample points. MZ twin pair correlations are plotted against those for DZ twins. The dashed line is the identity line. Error bars denote 95% confidence intervals derived through 10,000 bootstrap samples. D. Correlation difference estimates for population receptive field parameters in each visual region. Data are shown for polar angle, eccentricity, and pRF size parameters, as derived from pRF analysis. Filled circles indicate the group means. The violin plot shows the bootstrap distribution for each pRF property and visual region, and the error bars denote 95% confidence intervals. Asterisks indicate significant differences at p<0.05, after Bonferroni correction for multiple comparisons.
