# Supplementary material for: Heritable functional architecture in human visual cortex
Source: Neuroimage. 2021 Oct 1;239:118286. doi: 10.1016/j.neuroimage.2021.118286 (PMC7611349; doi:10.1016/j.neuroimage.2021.118286)
Supplement: Supplementary file 2 [file mmc2.pdf]

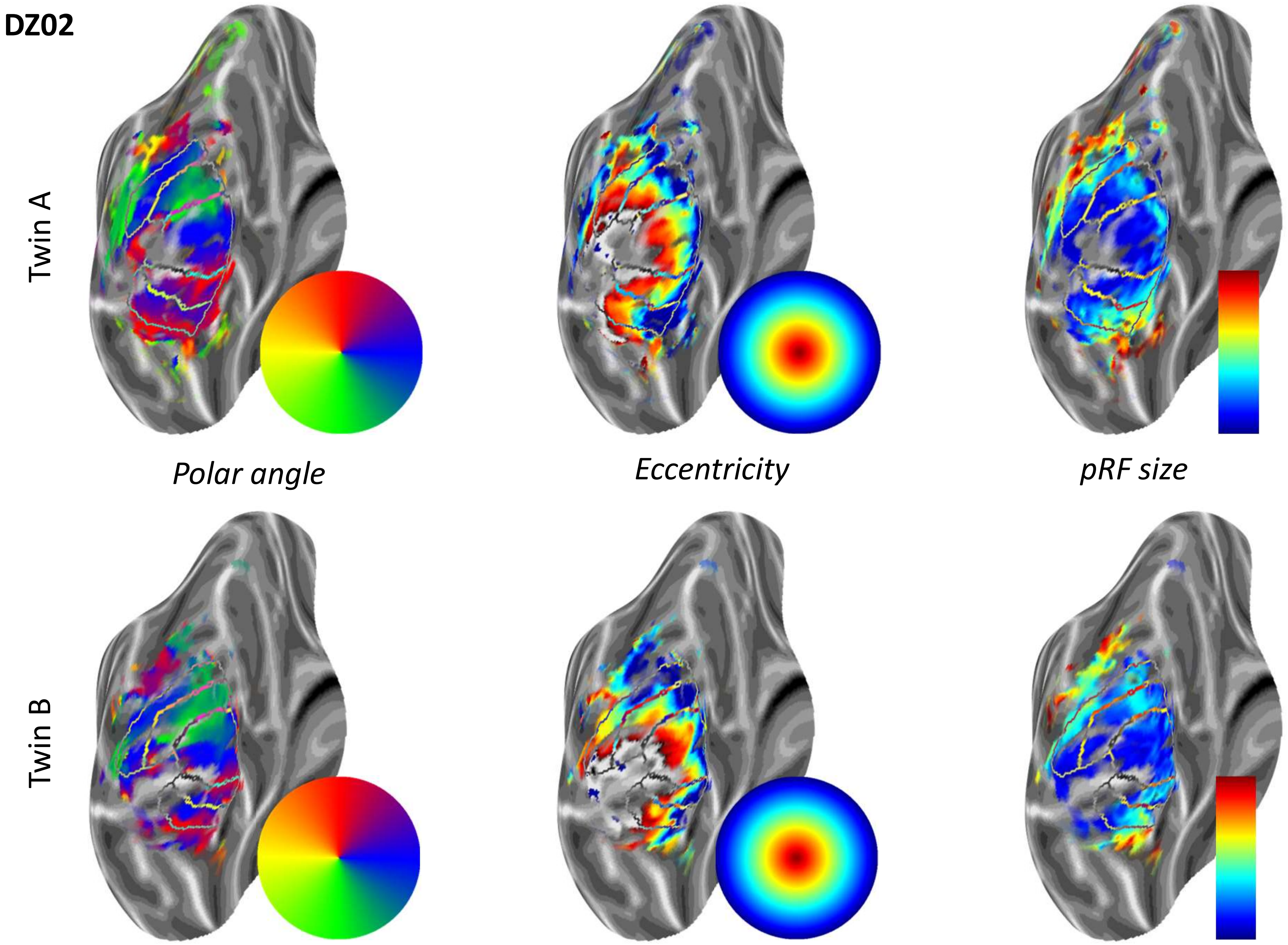
DZ02
Twin A
Twin B
Polar angle
Eccentricity
pRF size

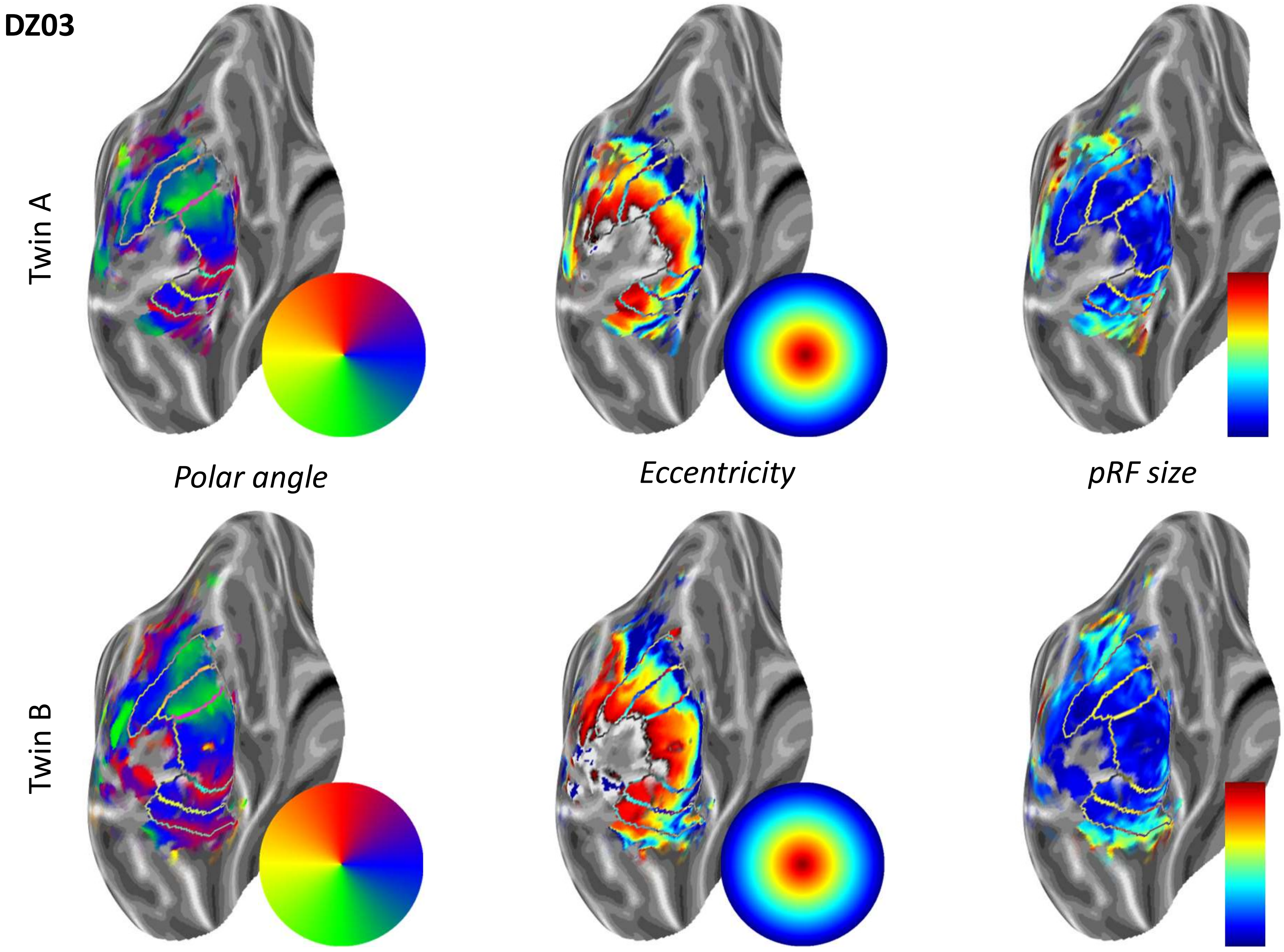
DZ03
Twin A
Twin B
Polar angle
Eccentricity
pRF size

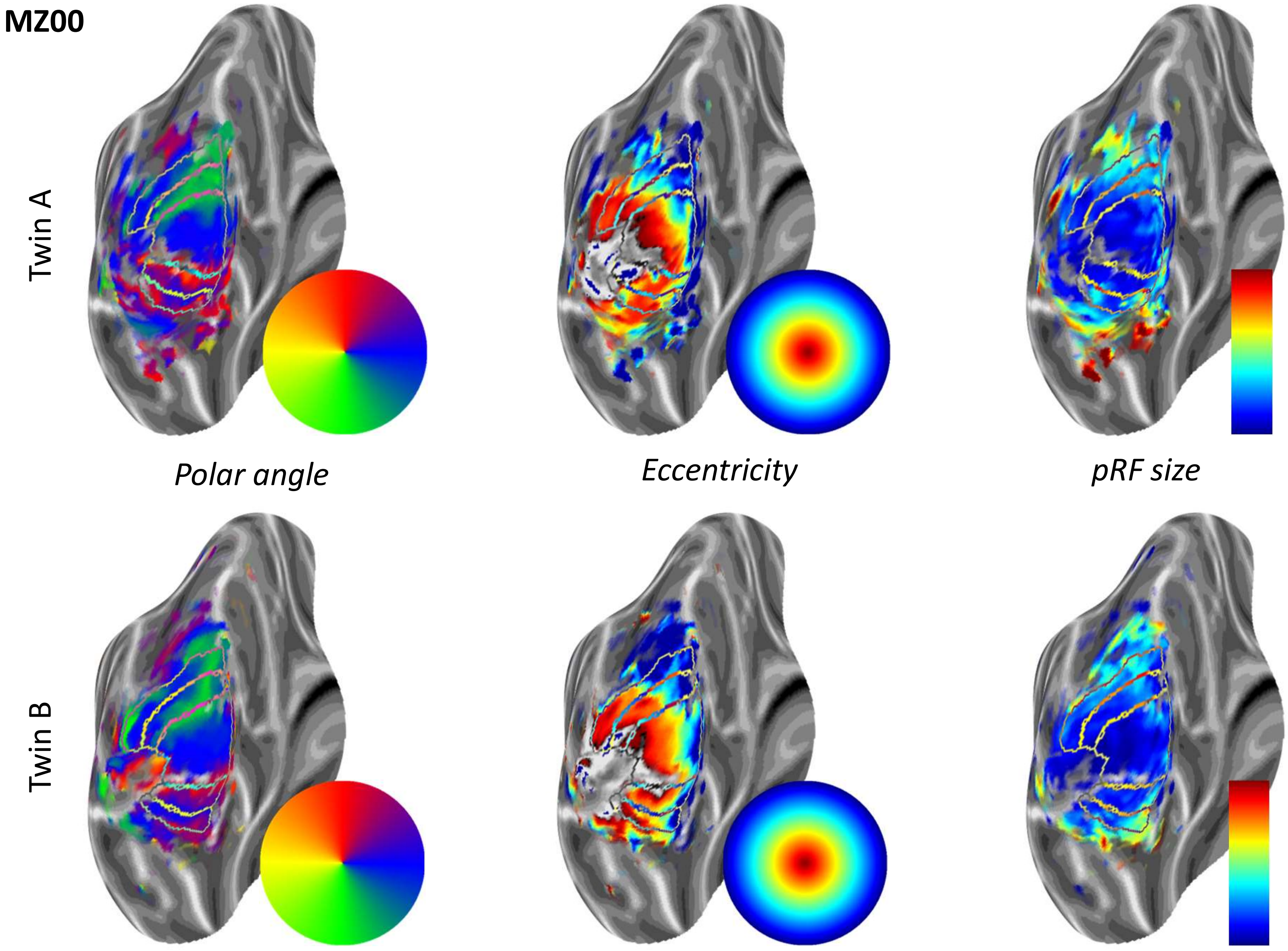
MZ00
Twin A
Polar angle
Eccentricity
pRF size
Twin B

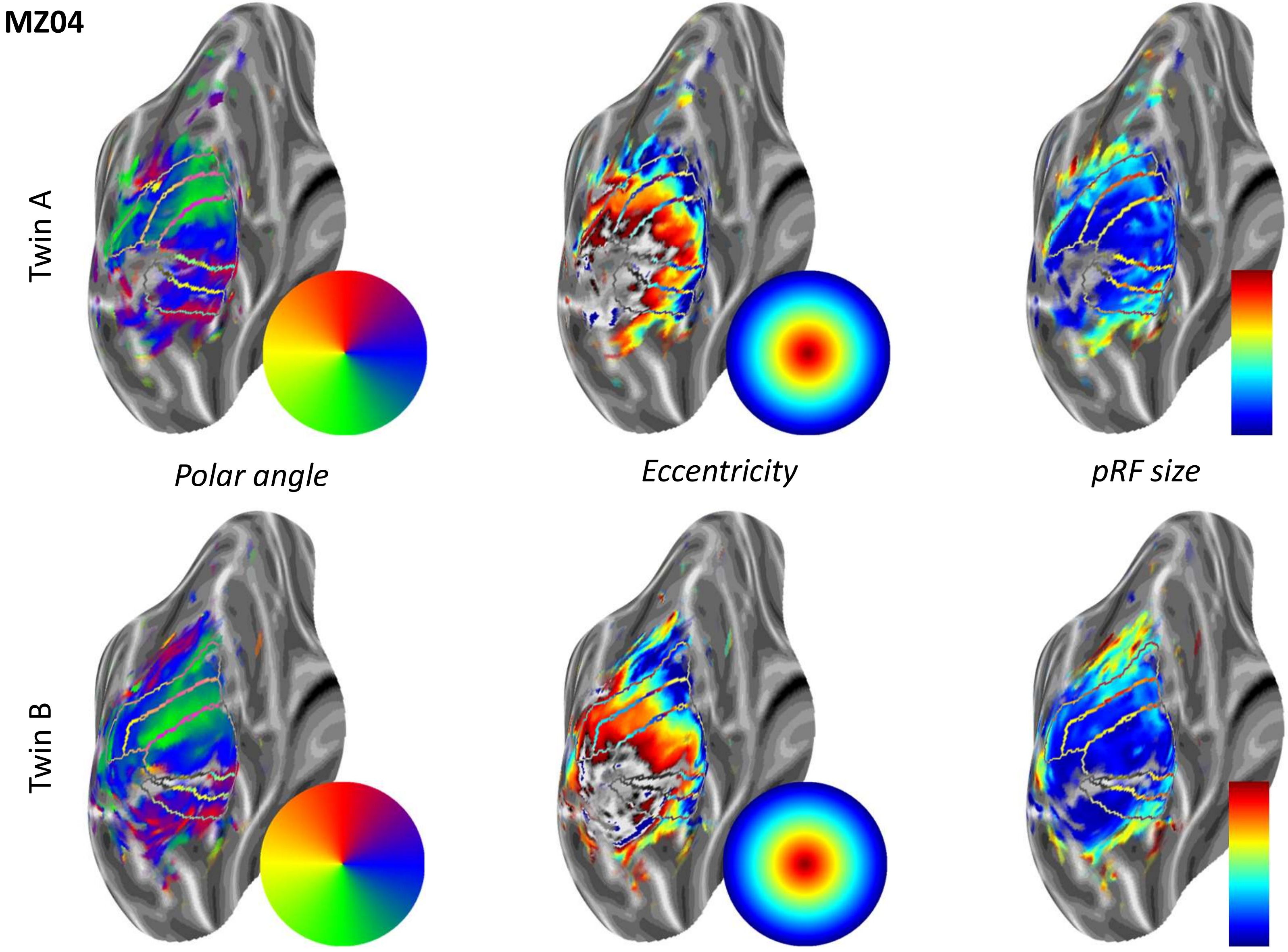
MZ04
Twin A
Polar angle
Eccentricity
pRF size
Twin B

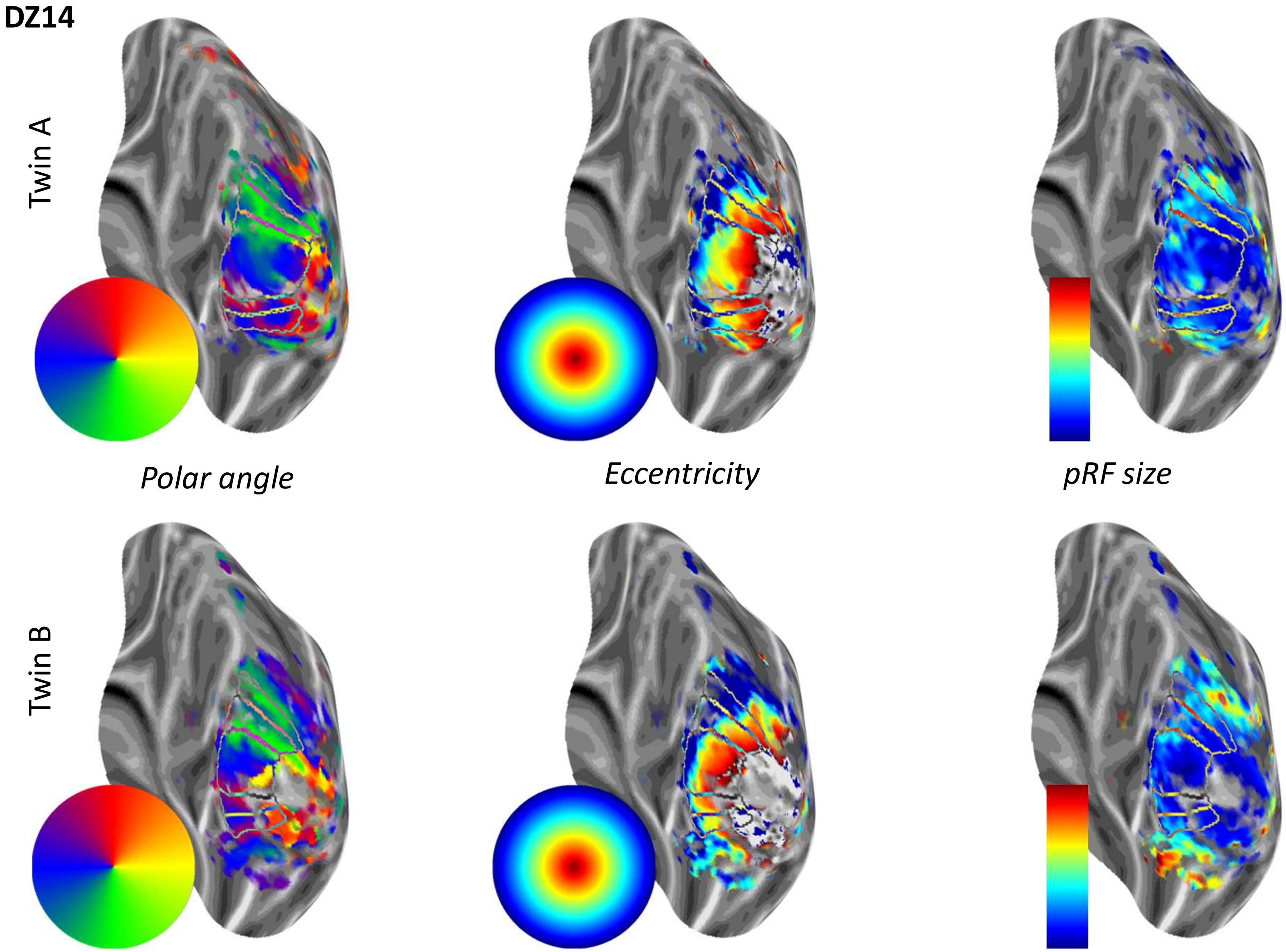
DZ14
Twin A
Polar angle
Eccentricity
pRF size
Twin B

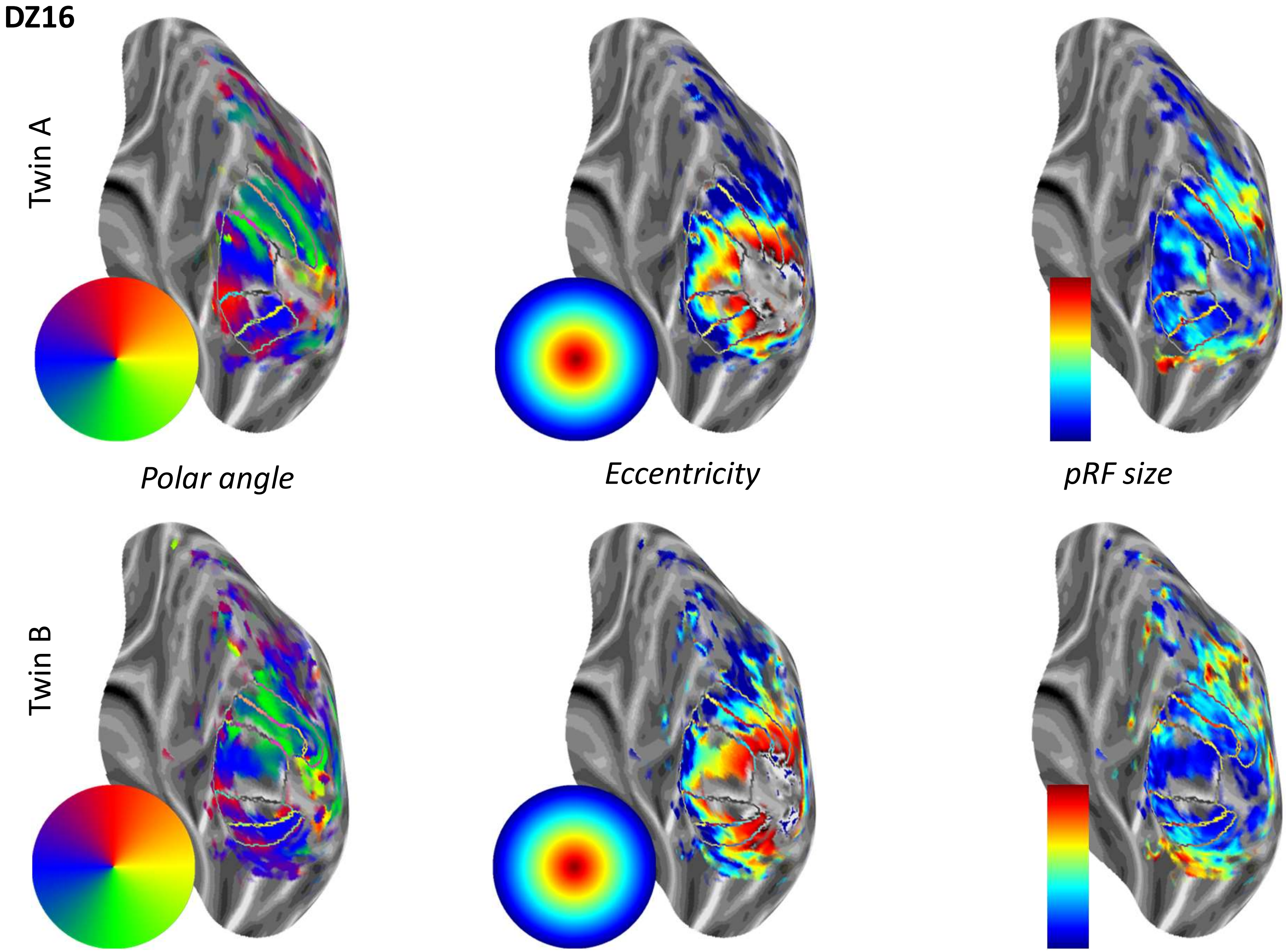
DZ16
Twin A
Polar angle
Eccentricity
pRF size
Twin B

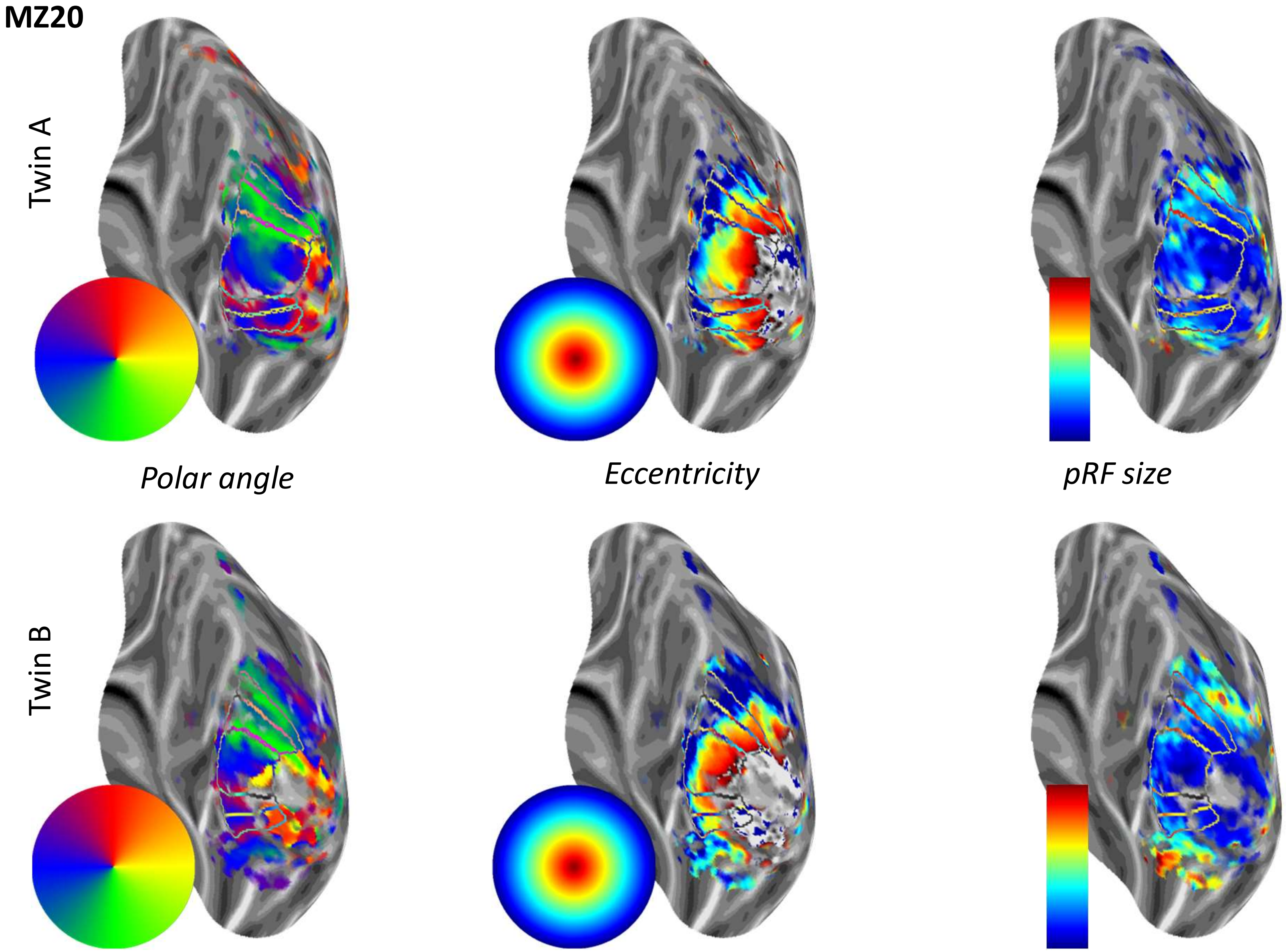
MZ20
Twin A
Polar angle
Eccentricity
pRF size
Twin B

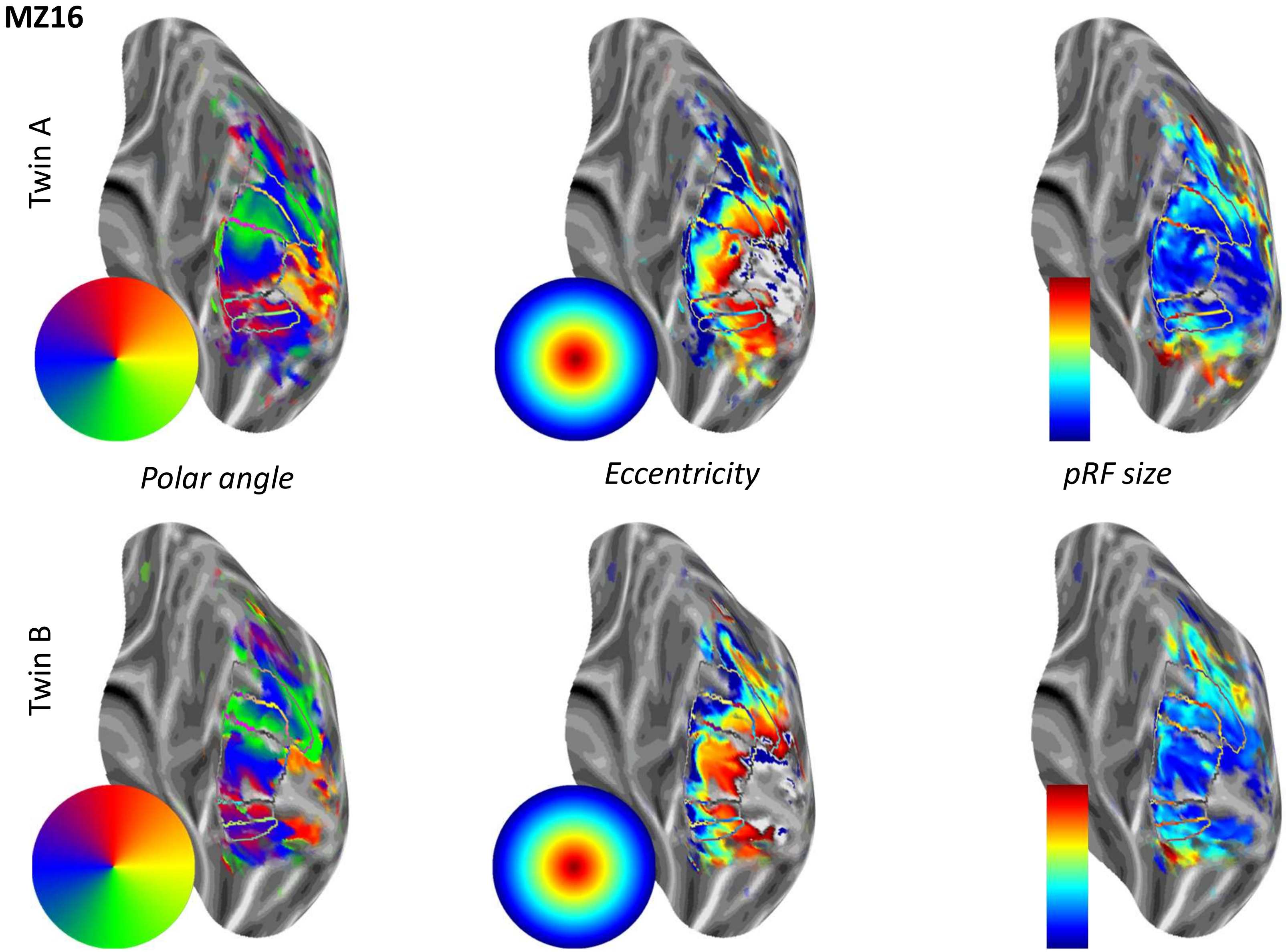
MZ16
Twin A
Polar angle
Eccentricity
pRF size
Twin B
