# Supplementary material for: Heritable functional architecture in human visual cortex
Source: Neuroimage. 2021 Oct 1;239:118286. doi: 10.1016/j.neuroimage.2021.118286 (PMC7611349; doi:10.1016/j.neuroimage.2021.118286)
Supplement: Supplementary file 3 [file mmc3.pdf]

Zygosity Questionnaire

1. My twin's name is: ________________________
2. What type of twin are you?
    a. Monozygotic (identical)
    b. Dizygotic (non-identical)
    c. Don't know
3. During childhood, were you and your twin as alike as 'two peas in a pod' or were you of ordinary family likeness?
    a. Like 'two peas in a pod'
    b. Of ordinary family likeness
    c. Don't know
4. Were you and your twin so similar in appearance at school age that people had difficulty in telling you apart?
    a. No
    b. Yes
    c. Don't remember
5. Who could tell you apart at school age?
    a. Parents (yes/no)
    b. Siblings other than twin (yes/no)
    c. School friends (yes/no)
    d. Strangers (yes/no)
6. At school, did you use any special mark or emblem to help tell you and your twin apart?
    a. No
    b. Yes
    c. Don't remember
7. How long did you live with your twin?
    a. I am still living with them
    b. We lived together until the age of ____.
8. Which of you was born first?
    a. My twin
    b. Myself
    c. Don't know
9. Are you one of triplets or quadruplets?
    a. No
    b. Yes
10. What is your education level?
    a. Less than high school
    b. High school
    c. Diploma
    d. Undergraduate
    e. Postgraduate
11. How much do you agree with this statement: "My twin and I often shared the same friends growing up"?

   - *a.* Strongly disagree
   - *b.* Disagree
   - *c.* Neither agree/disagree
   - *d.* Agree
   - *e.* Strongly agree

12. How much do you agree with this statement: “My twin and I often dressed alike growing up”?
   - *a.* Strongly disagree
   - *b.* Disagree
   - *c.* Neither agree/disagree
   - *d.* Agree
   - *e.* Strongly agree

13. How much do you agree with this statement: “My twin and I were usually in the same school classes *below* the age of 12”?
   - *a.* Strongly disagree
   - *b.* Disagree
   - *c.* Neither agree/disagree
   - *d.* Agree
   - *e.* Strongly agree

14. How much do you agree with this statement: “My twin and I were usually in the same school classes *above* the age of 12”?
   - *a.* Strongly disagree
   - *b.* Disagree
   - *c.* Neither agree/disagree
   - *d.* Agree
   - *e.* Strongly agree

15. How often have you seen your twin in person in the last year?
   - *a.* Not at all
   - *b.* Once or twice
   - *c.* 3-5 times
   - *d.* 6-10 times
   - *e.* 11-20 times
   - *f.* 20+ times

16. How often have you contacted your twin in the last year (e.g. by phone, email, fax, or letter)?
   - *a.* Not at all
   - *b.* Once or twice
   - *c.* Every few months
   - *d.* Monthly
   - *e.* Weekly
   - *f.* Daily

Please let the experimenter know if you have any further questions or information to provide.
